# The Pro-Apoptotic Role of the Regulatory Feedback Loop between miR-124 and PKM1/HNF4α in Colorectal Cancer Cells

**DOI:** 10.3390/ijms15034318

**Published:** 2014-03-11

**Authors:** Yan Sun, Xiaoping Zhao, Man Luo, Yuhong Zhou, Weiying Ren, Kefen Wu, Xi Li, Jiping Shen, Yu Hu

**Affiliations:** 1 Department of Geriatrics, Zhongshan Hospital, Fudan University, 180 Fenglin Road, Shanghai 200032, China; E-Mails: sunyansy00@163.com (Y.S.); luo.man@zs-hospital.sh.cn (M.L.); ren.weiying@zs-hospital.sh.cn (W.R.); wu.kefen@zs-hospital.sh.cn (K.W.); li.xi@zs-hospital.sh.cn (X.L.); shen.jiping@zs-hospital.sh.cn (J.S.); 2 Department of Nuclear Medicine, Ren Ji Hospital, School of Medicine, Shanghai Jiao Tong University, Shanghai 200127, China; E-Mail: xiaopingzhaoxp@gmail.com; 3 Department of Oncology, Zhongshan Hospital, Fudan University, Shanghai 200032, China; E-Mail: zhou.yuhong@zs-hospital.sh.cn

**Keywords:** PKM, HNF4α, miR-124, colorectal cancer

## Abstract

Accumulating evidence indicates that miRNA regulatory circuits play important roles in tumorigenesis. We previously reported that miR-124 is correlated with prognosis of colorectal cancer due to PKM-dependent regulation of glycolysis. However, the mechanism by which miR-124 regulates apoptosis in colorectal cancer remains largely elusive. Here, we show that miR-124 induced significant apoptosis in a panel of colorectal cancer cell lines. The mitochondrial apoptosis pathway was activated by miR-124. Furthermore, the pro-apoptotic role of miR-124 was dependent on the status of PKM1/2 level. PKM1 was required for miR-124-induced apoptosis. Via direct protein-protein interaction, PKM1 promoted HNF4α binding to the promoter region of miR-124 and transcribing miR-124. Moreover, HNF4α or PKM1 had a more dramatic effect on colorectal cancer cell apoptosis in the presence of miR-124. However, inhibition of miR-124 blocked cell apoptosis induced by HNF4α or PKM1. These data indicate that miR-124 not only alters the expression of genes involved in glucose metabolism but also stimulates cancer cell apoptosis. In addition, the positive feedback loop between miR-124 and PKM1/HNF4α plays an important role in colorectal cancer cell apoptosis; it suggests that disrupting this regulatory circuit might be a potential therapeutic tool for colorectal cancer treatment.

## 1. Introduction

Colorectal cancer (CRC) is the third most common malignancy worldwide. About 608,700 deaths from CRC occurred in 2008, accounting for 8% of all cancer deaths [1]. Deregulation of the apoptotic signaling pathway is one of the basic theories for tumorigenesis and progression of CRC [2,3]. Thus, a better understanding of the apoptosis-regulatory network will help to improve the clinical management of the disease and delay progression.

MicroRNAs (miRNAs) are single-stranded noncoding RNAs that silence their target genes by inhibiting messenger RNA (mRNA) translation or causing mRNA degradation [4]. Recent studies have implicated the involvement of miRNAs in many aspects of cancer development, including regulating the cell cycle, metabolism, and proliferation, as well as apoptosis [5]. In the past few years, accumulating evidence has suggested that microRNAs (miRNAs) are involved in the pathogenesis of CRC [6]. The pattern of miRNA expression is related with cancer type, stage and other clinical variables. miR-124 is commonly down-regulated in several types of cancer. Aberrant expression of miR-124 is related to methylation status of its locus [7]. In hepatocellular carcinoma, miR-124 is involved in an inflammatory feedback loop where it suppresses the expression of IL-6R and inhibits STAT3 activation. Systemic administration of miR-124 prevents hepatocellular carcinogenesis by inducing tumor-specific apoptosis [8]. In glioblastoma, miR-124 is implicated in T-cell-mediated antitumor immune response [9]. Our previous study found that miR-124 is significantly down-regulated in CRC patients with ≥5-year survival compared to patients with <5 year survival. The level of miR-124 is associated with poor prognosis of CRC [10]. Similar to our results, Zhang *et al.* also reported that miR-124 is significantly down-regulated in CRC tissue as compared to that of adjacent non-tumor colorectal tissue [11]. miR-124 is shown to directly suppress iASPP (inhibitor of apoptosis stimulating protein of p53) protein expression and up-regulate NF-κB level, which suppresses cell proliferation of CRC cells [12]. Over-expression of miR-124 leads to increased apoptosis of CRC cells and reduces tumor growth *in vitro* and *in vivo* [11]. However the mechanism underlying miR-124-induced apoptosis remains largely unknown.

Dysregulation of glucose metabolism is associated with multiple types of cancer. Pyruvate kinase (PK), which converts phosphoenolpyruvate to pyruvate, is one of the rate-limiting enzymes in glucose carbon flux. PK isoforms include liver (PKL) and red blood cells (PKR) [13]. The other two isoforms of PK are found in mammals: PKM1, which is expressed in adult tissue, and PKM2, which is expressed in embryonic tissue and tumors. PKM1 and PKM2 differ by only 23 amino acids within a 56-residue alternatively spliced exon (9 or 10, respectively) [14]. Three heterogeneous nuclear ribonucleoproteins (hnRNPs) proteins, including polypyrimidine tract binding protein (PTB, also known as hnRNPI), hnRNPA1 and hnRNPA2, bind repressively to sequences flanking exon 9. In the presence of the PKM alternative splicing proteins (PTB1, hnRNAPA1 and hnRNAPA2), exon 10 is included in the PKM transcript [15]. We have previously shown that PTB and HnRNAPA2 are downstream targets of miR-124, and that miR-124 switches PKM gene expression from PKM2 to PKM1 via PTB1/hnRNAPA1/hnRNAPA2-mediated PKM alternative splicing [10]. As a result, miR-124 shunts the glucose flux from glycolysis to oxidative phosphorylation, which could inhibit the growth of colorectal cancer cells. miR-124 not only has a role in apoptosis but also regulates glucose metabolism by switching *PKM* gene expression from *PKM2* to *PKM1*. However it is still unknown whether miR-124 could induce colorectal cancer cell apoptosis through modulating PKM alternative splicing.

HNF4α is a highly conserved member of the nuclear receptor superfamily of ligand dependent transcription factors [16]. It is expressed in the liver, pancreas, kidney, stomach, small intestine and colon, where it regulates many important aspects of cancer cell development. HNF4α is lost in the majority of colon cancer patients [17]. Hatziapostolou *et al.* [8] reported that miR-124 is a direct downstream target of HNF4α. ChIP analysis indicated that HNF4α strongly binds to the promoter region of miR-124. The HNF4α-miR124 circuit has important roles in tumorigenesis [8].

In this study, we show that miR-124 induced apparent apoptosis in a panel of CRC cell lines. The pro-apoptotic effect of miR-124 was dependent on the balance of PKM1/PKM2. Importantly, PKM1 as a cofactor of HNF4α was involved in miR-124 transcription. PKM1 or HNF4α regulating apoptosis of colorectal cancer cells was partially via miR-124. Overall our data suggests that a regulatory feedback loop between miR-124 and PKM1/HNF4α exists and is essential for apoptosis regulation in colorectal cancer cells.

## 2. Results and Discussion

### 2.1. miR-124 Regulates Apoptosis in Colorectal Cancer Cells

**Figure 1.**
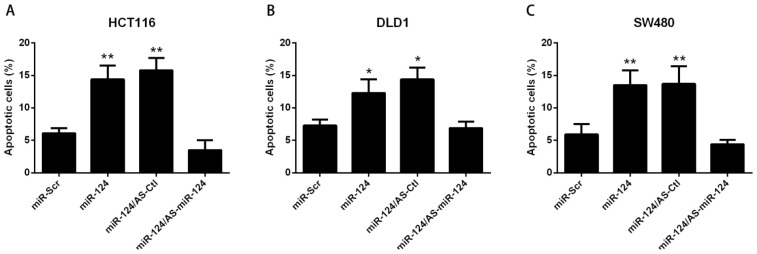

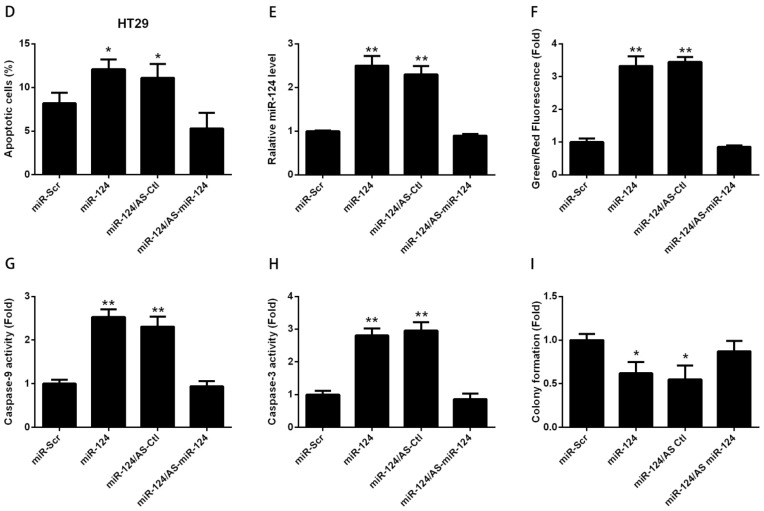
miR-124 regulates apoptosis in colorectal cancer cells. (**A**–**D**) DLD1, HCT116, SW480 or HT29 cells were transfected with miR-124 or antisense oligonucleotide (AS-miR-124), miR-Scr represented as the scrambled miRNA, and AS-Ctl represented the control of AS-miR-124 for 48 h. Apoptosis was assessed by Annexin V/PI assay; (**E**) miR-124 level was analyzed by real-time PCR, while HCT116 cells were transfected with miR-124 or AS-miR-124; (**F**,**G**) HCT116 cells were transfected with miR-124 or AS-miR-124, miR-Scr represented scrambled miR-124, AS-Ctl represented control of AS-miR-124 for 48 h; (**F**) Mitochondrial membrane potential was determined using JC-1. Loss of mitochondrial membrane potential was indicated by an increase in the green/red fluorescence intensity ratio. Fold-increase in green/red fluorescence was determined by comparing the results of treated samples with the level of the scrambled miRNA; (**G**) Caspase-9 activity or (**H**) Caspase-3 activity was analyzed by caspase-9 or caspase-3 Colorimetric Assay Kits respectively. Fold-increase in caspase-9 or caspase-3 activity was determined by comparing the results of treated samples with the level of the scrambled miRNA. The data are presented as mean ± SD of three independent experiments; (**I**) HCT-116 cells were seeded into six-well plates and treated with miR-124 or AS-miR-124. The fold-change of colony formation was determined by comparing the results of treated samples with the level of the scrambled miRNA. The data are presented as mean ± SD of three independent experiments. * *p* < 0.05 *vs.* control, ** *p* < 0.01 *vs.* control.

To investigate the biological effect of miR-124 in colorectal cancer cells, miR-124 and Scrambled miRNA (miR-Scr) were transfected into DLD1, HCT116, SW480 or HT29 cells respectively. As shown in Figure 1A–D, miR-124 apparently induced apoptosis in all colorectal cancer cell lines tested. To further validate the pro-apoptotic effect of miR-124, antisense oligonucleotide (AS-miR-124) was used as an inhibitor of miR-124. We found that AS-miR-124 repressed cell apoptosis induced by miR-124, supporting the suppressive role of miR-124 in colorectal cancer (Figure 1A–D). Further, miR124 level was measured to verify whether miR-124 was Over-expressed or silenced by the indicated treatment in HCT116 cells (Figure 1E). HCT116 cells had the most significant changes among all these colorectal cancer cells. Therefore, HCT116 cells were chosen as the representative colorectal cancer cells for further study.

Our previous report indicates that miR-124 regulates CRC cell proliferation via glucose metabolism regulation [10]. Mitochondrial organelle and Bcl-2 family proteins are the nexus of glucose metabolism and apoptosis signaling [18]. Therefore we hypothesized that the intrinsic apoptotic pathway was involved in miR-124-induced apoptosis. To determine that such increase in apoptosis was due to activation of the intrinsic apoptotic pathway, we tested mitochondrial membrane potential (MMP), activities of caspase-9 and caspase-3. Loss of mitochondrial membrane potential is an important indicator of cell intrinsic apoptosis. The fluorescent probe JC-1 has been shown to be most specific for measuring changes in the MMP. HCT116 transfected with miR-124 had a significant decrease in MMP as indicated by a notable increase in the ratio of green/red fluorescence *vs.* control. Conversely, miR-124-induced decrease in MMP can be reversed by AS-miR-124 (Figure 1F). Caspase-9 plays important roles in the process of mitochondria-mediated apoptosis. Caspase-3 is one of the downstream effectors of the caspase family, and it is involved in both the mitochondrial apoptotic pathway and the death receptor pathway. miR-124 promoted the activities of caspase-9 (Figure 1G) and caspase-3 (Figure 1H) as compared with control, which was significantly alleviated by AS-miR-124. Colony formation of HCT116 cells was increased by miR-124 (Figure 1I). Overall, these results suggest that the mitochondrial apoptotic pathway is involved in the miR-124-induced CRC cell apoptosis. These findings further support the therapeutic role of miR-124 in colorectal cancer treatment.

### 2.2. miR-124-Induced Apoptosis Is Dependent on the PKM Gene

**Figure 2.**
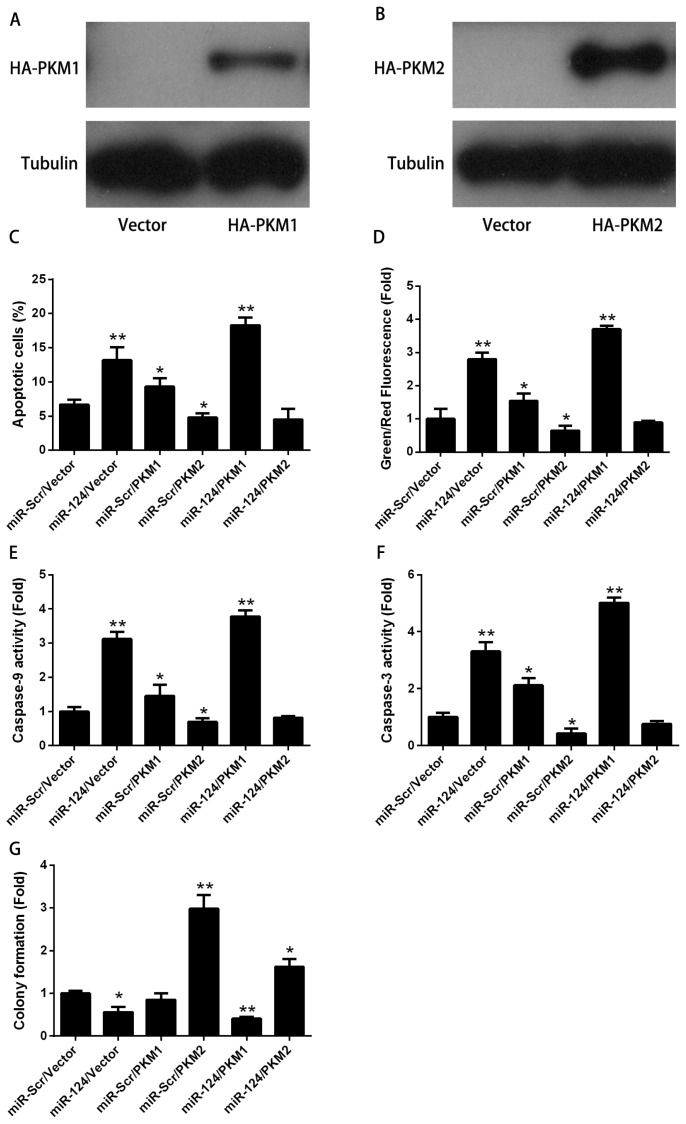
miR-124-induced apoptosis is dependent on the *PKM* gene. To investigate the involvement of PKM1 or PKM2 in the apoptosis induced by miR-124, HA tagged PKM1 (**A**) and HA tagged PKM2 (**B**) were used. HCT116 cells were cotransfected with miR-124 together with HA tagged PKM1 or HA tagged PKM2, miR-Scr represented the scrambled miRNA, and vector represented the empty plasmid for 48 h; (**C**) Apoptosis; (**D**) Mitochondrial membrane potential; (**E**) Caspase-9 activity; (**F**) Capase-3 activity; (**G**) Colony formation assay. The data are presented as mean ± SD of three independent experiments. * *p* < 0.05 *vs.* control, ** *p* < 0.01 *vs.* control.

Similar to other miRNAs, miR-124 also has multiple targets, including STAT3, SOS1, CD151 CDK4 and others [8,19,20]. Previously, we identified PTB and HnRNAPA2 as downstream targets of miR-124 [10]. PTB1, hnRNAPA1 and hnRNAPA2 control the alternative splicing of the *PKM* gene. The two isoforms, *PKM1* and *PKM2*, are produced from mutually exclusive alternative splicing of *PKM* gene. Cells preferentially have a high ratio of PKM2/PKM1 in the presence of PTB1, hnRNAPA1 and hnRNAPA2 [15]. The embryonic pyruvate kinase isoform, PKM2, is almost universally re-expressed in cancer. We have previously shown that miR-124 switches *PKM* gene expression from PKM2 to PKM1 via the PTB1/hnRNAPA2-mediated PKM alternative splicing [10]. Therefore, we asked whether the level of PKM2 or PKM1 was essential for miR-124-mediated apoptosis. To investigate the involvement of PKM1 or PKM2 in apoptosis induced by miR-124, HA tagged PKM1 (Figure 2A) and HA tagged PKM2 (Figure 2B) were used. As shown in Figure 2C, PKM2 significantly attenuated the increase of apoptosis of HCT116 cells which was evoked by miR-124. Consistently, the changes of MMP (Figure 2D), caspase-9 activity (Figure 2E) and caspase-3 activity (Figure 2F) were compromised in the presence of PKM2. On the other hand, the pro-apoptotic effect of miR-124 was exaggerated in the presence of PKM1 (Figure 2C). Simultaneous treatment with miR-124 and PKM1 enhanced the effect of miR-124 on activation of the mitochondrial apoptotic pathway, as indicated by MMP (Figure 2D), caspase-9 activity (Figure 2E) and caspase-3 activity (Figure 2F). These results suggest that PKM1 exaggerates the pro-apoptotic effect of miR-124. The combination of miR-124 with PKM1 significantly decreased the colony formation of HCT116 cells consistently. Colony formation induced by PKM2 was down-regulated in the presence of miR-124 (Figure 2G). The changes of the intrinsic apoptotic pathway, including MMP and caspase activity, support that PKM1/2 status is essential for the role of miR-124 in apoptosis regulation.

### 2.3. PKM1 Regulates miR-124 Expression via HNF4α

**Figure 3.**
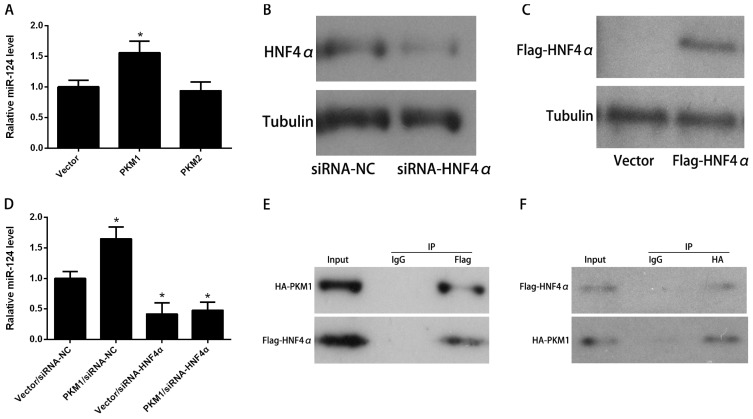

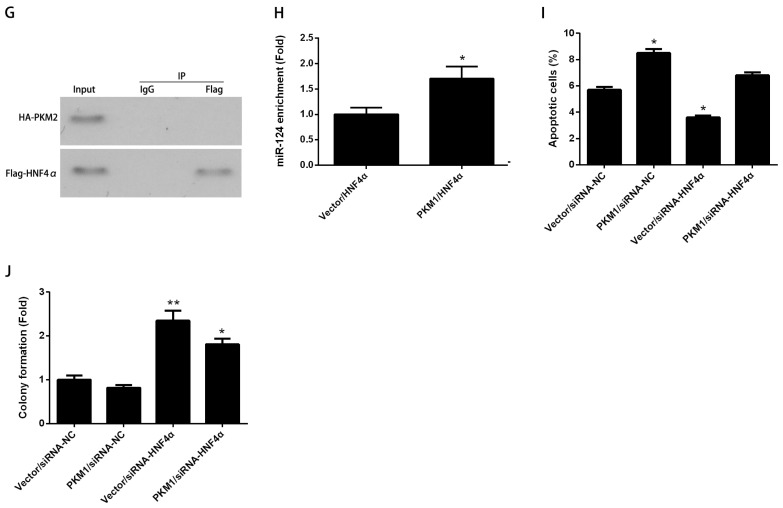
PKM1 regulates miR-124 expression via HNF4α. (**A**) To test whether PKM1 could regulate miR-124 expression, HCT116 cells were transfected with HA-PKM1 or empty vector for 48 h. Cells were then collected for analyzing miR-124 levels; To address the potential involvement of HNF4α in induction of miR-124 by PKM1; (**B**) HNF4α was silenced by specific siRNA; and (**C**) Flag-tagged HNF4α was used for over-expression; (**D**) HCT116 cells were cotransfected with the siRNA control (siRNA-NC) or siRNA against HNF4α (siRNA HNF4α) together with empty vector or HA-PKM1 for 48 h. miR-124 level was analyzed by real-time PCR; (**E**–**G**) Interaction between Flag-HNF4α and HA-PKM1 or HA-PKM2 was examined by co-immunoprecipitation. IgG or Flag antibody was used for immunoprecipitation, followed by Western blotting using anti-HA antibody; (**H**) To address the potential role of PKM1 in induction of miR-124 by HNF4α, HCT116 cells were cotransfected with Flag-HNF4α together with HA-PKM1 or empty vector for 48 h, cells were immunoprecipitated by Flag antibody. Real-time PCR was applied for analyzing the miR-124 enrichment; (**I**) To determine whether PKM1-induced apoptosis depended on HNF4α, HCT116 cells were cotransfected with siRNA-NC or siRNA HNF4α together with empty vector or HA-PKM1 for 48 h. Apoptosis was analyzed by Annexin V/PI assay; (**J**) HCT116 cells cotransfected with siRNA control (siRNA-NC) or siRNA against HNF4α (siRNA HNF4α) together with empty vector or HA-PKM1. The fold-change of colony formation was determined by comparing the results of treated samples with the level of the vector/siRNA-NC control. The data are presented as mean ± SD of three independent experiments. * *p* < 0.05 *vs.* control, ** *p* < 0.01 *vs.* control.

As mentioned above, miR-124 had more dramatic effect on apoptosis in the presence of PKM1 (Figure 2). The synergistic role of miR-124 and PKM1 prompted us to study their relationship in apoptosis regulation. Since miRNA-mediated regulation frequently contains regulatory feedback loops [21,22], we hypothesized that PKM1 was probably an upstream regulator of miR-124. We observed that over-expression of PKM1 but not PKM2 resulted in a significant increase of miR-124 level (Figure 3A). Unfortunately, ChIP assay did not find PKM1 directly interacting with the promoter region of miR-124 (data not shown). Therefore we hypothesized that PKM1 had an indirect role in regulating miR-124 expression. It has been previously shown that miR-124 is a direct downstream effector of HNF4α [8]. To address the potential involvement of HNF4α in induction of miR-124 by PKM1, HNF4α was silenced by specific siRNA (Figure 3B) and Flag-tagged HNF4α was used for over-expression (Figure 3C). Consistently, our study showed that HNF4α knockdown decreased the level of miR-124. In the absence of HNF4α, the expression of miR-124 induced by PKM1 was completely abolished (Figure 3D). It suggests that PKM1-induced miR-124 expression is dependent on HNF4α. Several lines of evidence support the contention that pyruvate kinase had non-catalytic roles via protein–protein interaction. PKM2 has been found as a cofactor of HIF1α to induce the expression of genes involved in glucose metabolism of cancer cells [23]. Likewise, PKM2 directly binds to histone H3 and phosphorylates histone H3, which is required for the transcription of CCND1 and MYC [24]. Here we found that Flag-HNF4α could pulldown HA-PKM1 from the cell lysates of HCT116 cells (Figure 3E). HA-PKM1 also immunoprecipitated with Flag-HNF4α in HCT116 cells (Figure 3F). Interestingly, there was no interaction between HA-PKM2 and Flag-HNF4α in HCT116 cells (Figure 3G). Moreover, the binding of HNF4α to the promoter region of miR-124 was significantly increased by PKM1 (Figure 3H). HNF4α was also required for PKM1-induced apoptosis (Figure 3I). In the presence of PKM1, the effect of *HNF4α* knockdown on colony formation was inhibited (Figure 3J). These results indicate that PKM1 promotes miR-124 expression via direct interaction with HNF4α; PKM1 is thus likely a cofactor with HNF4α to induce miR-124 expression.

### 2.4. PKM1/HNF4α Regulates Apoptosis via miR-124

**Figure 4.**
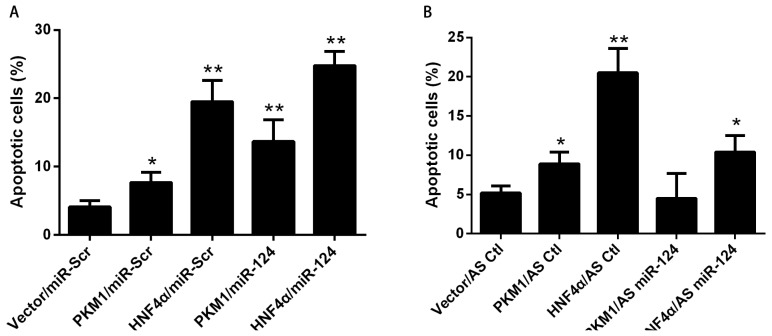

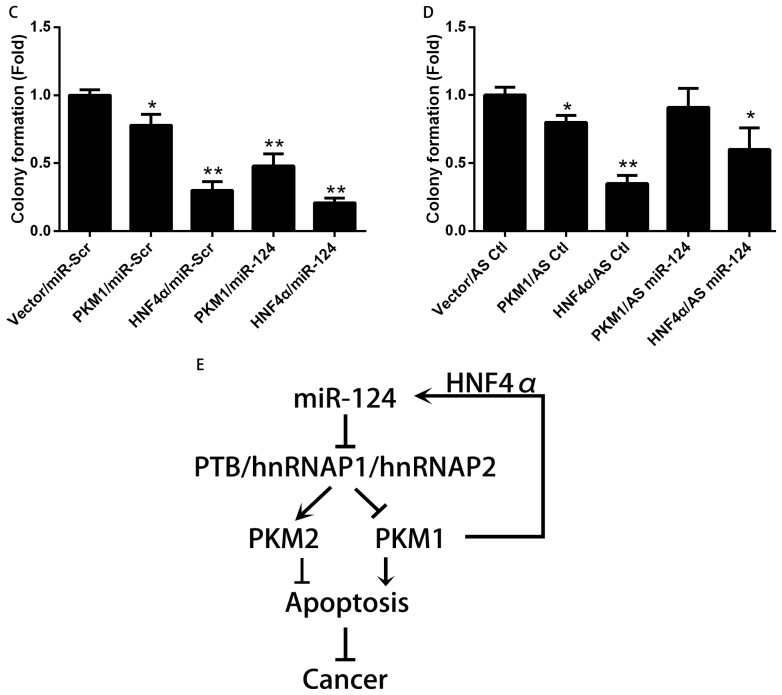
PKM1/HNF4α regulates apoptosis via miR-124. To determine the role of miR-124 in PKM1/HNF4α-induced apoptosis, (**A**) HCT116 cells were cotransfected with HA-PKM1, Flag-HNF4α or empty plasmid (vector) together with miR-124 or miR-Scr for 48 h; (**B**) HCT116 cells were cotransfected with HA-PKM1, Flag-HNF4α or empty plasmid (vector) together with AS-miR-124 or AS-Ctl for 48 h; (**A**,**B**) Apoptosis was analyzed by Annexin V/PI assay; (**C**,**D**) The anchorage-independent cell growth was analyzed by colony formation assay; (**E**) Working model, in summary, MiR-124 regulates the ratio of PKM1/PKM2 via targeting PKM splicing proteins (PTB/hnRNAP1/hnRNAP2). The ratio of PKM1/PKM2 determines the apoptosis rate of cancer cells. In the meantime, PKM1 exerts positive feedback regulation on miR-124 level through HNF4α transcription factor. The data are presented as mean ± SD of three independent experiments. * *p* < 0.05 *vs.* control, ** *p* < 0.01 *vs.* control.

We next tested whether the positive feedback between PKM1/HNF4α and miR-124 had an important role in cell apoptosis. As shown in Figure 4A, either PKM1 or HNF4α induced apoptosis in HCT116 cells. Furthermore, miR-124 enhanced the pro-apoptotic effect of PKM1 on HCT116 cells. The effect of HNF4α on apoptosis was also increased in the presence of miR-124 (Figure 4A). To further validate the role of miR-124 in PKM1/HNF4α-mediated apoptosis, miR-124 dependency was analyzed. As shown in Figure 4B, apoptosis induced by PKM1 or HNF4α was partially inhibited by AS-miR-124. To verify whether the positive feedback between PKM1/HNF4α and miR-124 was involved in the colony formation capacity of HCT 116 cells, anchorage-independent cell growth was analyzed. As shown in Figure 4C, there was a synergistic effect between miR-124 and PKM1 in inhibiting colony formation of HCT 116 cells. Similar phenotypes were observed in cells treated with both miR-124 and HNF4α. Interestingly, the inhibition effect of PKM1 or HNF4α on colony formation was slightly rescued by miR-124 knockdown (Figure 4D). Thus, these results indicate that the regulatory network of miR-124 and PKM1/HNF4α has a more drastic effect on cell apoptosis. The positive feedback between PKM1/HNF4α and miR-124 might play an important role in colorectal tumorigenesis (Figure 4E).

## 3. Experimental Section

### 3.1. Cell Lines and Cell Culture

The colorectal cell lines HCT116, DLD1, SW480 and HT29, were purchased from ATCC (Manassas, VA, USA). All cells were incubated at 37 °C in a humidified chamber supplemented with 5% CO_2_, using recommended medium and 10% FBS.

### 3.2. Transfection

All siRNAs, miRNA mimics, and miRNA inhibitors were purchased from Open Biosystem (Pittsburgh, PA, USA) and transfected into cells using Lipofectamine 2000 (Invitrogen, Carlsbad, CA, USA) According to the manufacturer’s instruction, cells were seeded into 6-, 24- or 96-well plate. After overnight incubation, cells were transfected with the indicated treatment for 4–6 h in Opti-MEM medium (Invitrogen, Carlsbad, CA, USA), then the culture medium was changed to regular medium for another 36–48 h and the cells were collected for related assays.

### 3.3. Apoptosis Analysis

Cell apoptosis was assayed by Annexin V-FITC Apoptosis Detection Kit (Beyotime Institute of Biotechnology, Shanghai, China). Following treatment, cells were harvested by treating with trypsin. Total cells were washed with cold phosphate-buffered saline (PBS) and resuspended in the annexin V binding buffer. A single-cell suspension was stained with 5 μL annexin V-FITC for 10 min at room temperature in the dark, and then incubated with PI for 2 min. Then cells were analyzed by flow cytometry [25].

### 3.4. Measurement of Mitochondrial Membrane Potential

Loss of mitochondrial membrane potential is an important indicator of cell apoptosis. The changes in mitochondrial membrane potential were measured by the JC-1 probe (Invitrogen, Carlsbad, CA, USA), which was dispersed from aggregated form (red fluorescence) to the monomeric form (green fluorescence) when mitochondrial membrane potential was lost. Loss of mitochondrial membrane potential was indicated by an increase in the green/red fluorescence intensity ratio. Cells were incubated with 5 μg/mL of JC-1 at 37 °C for 15 min and then analyzed by fluorescence-activated cell sorter [26].

### 3.5. Analysis of Caspase-3 and Caspase-9 Activities

Caspase-3 and caspase-9 activities were measured using Colorimetric Assay Kits (BioVision, Milpitas, CA, USA) according to the manufacturer’s instructions respectively. Harvested cells were resuspended in 50 μL of chilled cell lysis buffer and incubated on ice for 10 min. After they were centrifuged, the supernatant was transferred to a fresh tube and put on ice. Protein concentration was then assayed, and 100 μg protein was diluted to 50 μL cell lysis buffer for each assay. Protein was added in 5 μL of the 4 mM LEHD-pNA or DEVD-pNA substrate (200 μM final conc.; LEHD-pNA or DEVD-pNA for caspase-9 or caspase-3, respectively) and incubated at 37 °C for 1.5 h. Samples were measured at 405 nm in a microtiter plate reader. Fold-increase in caspase-9 or caspase-3 activity was determined by comparing the results of treated samples with the level of the control.

### 3.6. Real-Time PCR

For microRNA, we used stem-loop quantitative RT-PCR. Briefly, 25 ng of total RNA was reversely transcribed using the miRCURY First-strand cDNA kit and the miRCURY microRNA primer sets. QPCR was performed with the Sequence Detection System 7900HT (Applied Biosystems, Foster City, CA, USA) using the miRCURY LNA™ SYBR^®^ Green Master mix. The comparative *C*_t_ (ΔΔ*C*_t_) method was used to determine the expression level of miRNA and U6 as endogenous controls [27]. The primers used were as follows: *miR-124*, 5′-GCGGCGGTAAGGCACGCGGTT-3′ (forward) and 5′-GTGCAGGGTCCGAGGTATTCG-3′ (reverse); *U6*, 5′-TGCTCGCTTCGGCAGCACAT-3′ (forward) and 5′-CTTGCGCAGGGGCCATGCTA-3′ (reverse).

### 3.7. Western Blotting

After treatment at the indicated time, cells were harvested, and the proteins from cells were separated by SDS–PAGE in 12% (*w*/*v*) polyacrylamide gels and transferred to PVDF membranes. The membranes were then incubated with the appropriate primary antibody overnight at 4 °C. After subsequently rinsing in PBS, membranes were incubated with secondary antibody conjugated to horseradish peroxidase, the bands were visualized by enhanced chemiluminescence (Thermo Fisher Scientific, San Jose, CA, USA), and the blots were exposed to film (Thermo Fisher Scientific, San Jose, CA, USA) [28]. Flag antibody (Sigma, St. Louis, MO, USA), HA antibody (Abcam, Cambridge, MA, USA), PKM2 antibody (Cell Signaling Technology, Danvers, MA, USA), HNF4α antibody (Santa Cruz, CA, USA) were used for protein assay.

### 3.8. Chromatin Immunoprecipitation Assay

Chromatin immunoprecipitation (ChIP) was performed using the EZ-ChIP Assay kit (Millipore, Bedford, MA, USA) with an antibody to HNF4α (Abcam, Cambridge, MA, USA). Briefly, treated colorectal cancer cells were cultured in 150 mm culture dishes. The cells were then cross-linked with 1% formaldehyde for 10 min at room temperature, and the reaction was quenched with 0.125 M glycine. The cells were scraped, resuspended in lysis buffer (1% SDS, 10 mM EDTA, and 50 mM Tris–HCl, pH 8.1) supplemented with a protease inhibitor cocktail on ice, and sonicated to obtain DNA fragments of 200–1000 bp in length, as confirmed by electrophoresis on 1% agarose gels. The sheared DNA was centrifuged at 12,000× *g* at 4 °C for 10 min, and the supernatant was collected. Soluble chromatin was immunoprecipitated overnight with an anti-HNF4α antibody. The immunoprecipitated chromatin complex was harvested using protein G-agarose beads, and the crosslink was reversed by adding NaCl to a final concentration of 200 mM at 65 °C for 5 h. The DNA was purified using the spin columns provided with the kit. The DNA samples, as well as the input material and the mock immunoprecipitation samples, were used as templates for semi-quantitative and real time PCR to determine the relative enrichment of the miR-124 promoter.

### 3.9. Co-Immunoprecipitation

For immunoprecipitation, 200 μg of cell lysate were incubated with 25 μL each of agarose A and G (Invitrogen, Carlsbad, CA, USA) in 500 μL total volumes for 1 h. Immunoprecipitation antibody (Flag) was added with an additional 3 h of incubation at 4 °C with constant rotation. The complex was washed three times. It was then resuspended in SDS loading buffer. After boiling, the supernatant was loaded for western blot analysis. The total protein in the input lysate was approximately 1/10 of the amount used for immunoprecipitation. The HA antibody was use for western blot assay.

### 3.10. Colony Formation Assay

Cells transfected with indicated siRNA or miRNA were seeded at 1 × 10^4^ per dish and allowed to grow for an additional two weeks. The colonies were then fixed with methanol and stained with Crystal Violet (Sigma, St. Louis, MO, USA) and counted under a microscope.

### 3.11. Statistical Analysis

All experiments were performed in triplicate and the results are expressed as the means ± SD. The data were analyzed by the Student’s *t*-test or by the one-way analysis of variance (ANOVA), and *p* ≤ 0.05 denoted a statistically significant difference.

## 4. Conclusions

Taken together, our data support the model shown in Figure 4C. Briefly, miR-124 activates the mitochondrial apoptosis pathway via targeting the proteins involved in the regulation of PKM alternative splicing. The PKM gene is essential for miR-124-mediated apoptosis. Moreover, PKM1 facilitates HNF4α binding to the promoter region of miR-124 via direct protein-protein interaction and promotes the transcription of miR-124. The positive feedback regulation between miR-124 and PKM1/HNF4α causes significant apoptosis in CRC cells. We propose a hypothetical model for the regulation of CRC cells apoptosis by miR-124. In normal colorectal cells, miR-124 switches *PKM* gene expression from PKM2 to PKM1 via PTB1/hnRNAPA2-mediated PKM alternative splicing, resulting in metabolizing glucose via oxidative phosphorylation. Meanwhile, PKM1 increases the transcription of miR-124 by promoting HNF4α binding to the promoter region of miR-124. This positive feedback regulation prevents CRC tumorigenesis by inducing apoptosis. In contrary, loss of miR-124 increases the expression of PKM2 and decreases the expression of PKM1, which shunts glucose metabolism from oxidative phosphorylation to glycolysis to support cancer cell growth. At the same time, loss of PKM1 and miR-124 breaks the feed forward loop, consequently resulting in loss of abnormal colorectal cells apoptosis. This scenario may likely explain why both miR-124 and PKM1 were lost in tumorigenesis. Our results suggest that miR-124 and PKM1 promote mutually by forming positive feedback regulation, and act at the interface of cancer apoptosis and glucose metabolism. Therefore, treatment with miR-124 and PKM1 would be an effective therapeutic strategy for cancer.
